# Identification of hub genes associated with neutrophils infiltration in colorectal cancer

**DOI:** 10.1111/jcmm.16414

**Published:** 2021-03-05

**Authors:** Hao Su, Tianyi Cai, Sen Zhang, Xialin Yan, Leqi Zhou, Zirui He, Pei Xue, Jianwen Li, Minhua Zheng, Xiao Yang, Bo Feng

**Affiliations:** ^1^ Ruijin Hospital Shanghai Jiao Tong University School of Medicine Shanghai China; ^2^ Department of General Surgery Zhongshan Hospital, Fudan University Shanghai China; ^3^ Department of General Surgery Tongji University Tenth People's Hospital Shanghai China; ^4^ Department of General Surgery Changhai Hospital Shanghai China

**Keywords:** CIBERSORTx, colorectal cancer, immune cells, neutrophils, tumour microenvironment, WGCNA

## Abstract

Colorectal cancer (CRC) is the leading cause of cancer‐related mortality in the world. Accumulating evidence indicate that tumour infiltrating immune cells participated in cancer progression. Among them, tumour infiltrating neutrophils (TINs) are reported to play crucial role in various cancers. In this study, we used CIBERSORTx, a digital cytometry tool to evaluate the neutrophils infiltration in CRC based on gene expression data of CRC tissues from GSE39582 data set and The Cancer Genome Atlas data set (TCGA‐COAD and TCGA‐READ). Weighted gene co‐expression network analysis (WGCNA) was conducted in GSE39582 data set to identify hub genes associated with neutrophil infiltration. The association of hub gene and neutrophils was then validated in TCGA cohorts and an independent RJ cohort. Functional analysis was performed to investigate the molecular mechanisms of the interested hub gene. We found that neutrophil infiltration is elevated in CRC tissues, and it is related to a poorer prognosis. A total of 18 gene modules are identified by WGCNA in GSE39582 data set, among which lightcyan module is significantly correlated with neutrophils infiltration. Furthermore, Superoxide Dismutase 2 (SOD2) in lightcyan module was proved to correlated with neutrophils infiltration in various cancer types. In addition, SOD2 expression is highly associated with several chemokines, including CXCL8, a neutrophils‐related attractant, and functional analysis revealed that SOD2 is involved in neutrophils recruitment biological process. These results indicate that an ‘SOD2‐CXCL8‐neutrophil recruitment’ axis plays a potential role in colorectal cancer progression.

## 1 INTRODUCTION

Accounting for 1 in 10 people suffering from cancer, colorectal cancer (CRC) ranks as the third in terms of cancer incidence and second of in terms of mortality in the world. In 2018, Over 1.8 million new cases are identified, and approximately 881,000 people died of CRC.^1^ Despite the improvements of treatment methods, mainly surgical resection and chemotherapy, patients with advanced CRC are still haunted by the recurrence after surgery, which compromise life quality and expectance.^2^ In this context, a better understanding of CRC mechanism is needed to develop novel cures.

In recent years, the success of checkpoint‐blockade therapy in clinical trials shed light on the critical role of tumour infiltrating immune cells, especially T cells.^3^ As a historical milestone of cancer treatment, checkpoint blockade immunotherapies proved that our immune system can be harnessed to antagonize malignant tumour. However, despite these substantial advancements in clinical care, the majority of patients do not respond to immune checkpoint inhibitor.^4^

Increasing evidence suggests that tumorigenesis is often triggered in a tumour microenvironment (TME), which is composed of extracellular matrix, blood or lymphatic vessels, fibroblasts, immune cells and inflammatory cells. Importantly, the role of the crosstalk between tumour cells and the stromal cells in the process of tumour progression, drug resistance and clinical outcomes has been validated by multiple studies.^5^ As shown, various types of myeloid cells, such as tumour‐associated macrophages (TAMs), tumour‐infiltrated neutrophils (TINs) and T cells, play important roles in the above biological processes. Infiltration of cytotoxic CD8 + T cells has been associated with a favourable prognosis of CRC patients, mainly because of the anti‐tumour activity of CD8 + T cells. Gastric cancer patients with higher TINs were prone to overall survival benefit from postoperative adjuvant chemotherapy. A number of studies have shown that high levels of circulating neutrophils and neutrophil‐to‐lymphocyte ratio in peripheral blood are associated with poor prognosis in several types of cancers. However, the clinical significance of TINs in tumour stromal tissues is controversial.^6^

Activated neutrophils can release multiple toxic components, including MPO, H2O2, cationic proteins defensins and NET to kill the invaders. However, in the context of tumour, neutrophils can be manipulated by tumour cells and switch into pro‐tumour status.^7^ Emerging evidence indicates that neutrophil plays a contradictory role in TME. Other than the cytotoxicity mentioned above, it can degrade extracellular matrix by secretion of MMPs and elastase, facilitating tumour cells dissemination. It also promotes angiogenesis mediated by releasing VEGF, HGF and MMP9. Moreover, neutrophil mediates immune suppression via the secretion of ROS and Arginase 1 to limit T cell–dependent anti‐tumour immunity.^7^, ^8^ These pro‐tumour effects might be the potential therapeutic target in the treatment of cancer.

Plenty of studies described the phenomenon of neutrophils recruitment to inflammation or invasion spot by robust chemotactic molecules, most importantly CXCL1, CXCL2, CXCL5 and CXCL8.^9^ Tumours orchestrate neutrophils recruitment by releasing neutrophil‐related chemokines. For example, TME hypoxia induced CXCL8 overexpression through hypoxia inducible factor 1(HIF1) signalling pathway.^10^ However, different tumours may allure neutrophils in various ways, and little research work has been done to investigate the molecular mechanisms of interaction between colorectal cancer and neutrophils. In this research, we use bioinformatic tools to identify the genes potentially associated with neutrophil infiltration in colorectal cancer. Moreover, the interested hub gene was validated in CRC cases from TCGA cohort and real‐world validation cohort.

## 2 MATERIALS AND METHODS

### 2.1 Patients and samples

Gene expression data of 9164 sample of 32 types of cancer (including 603 colorectal cancer patients, COAD and READ) from TCGA were obtained from Xena (https://xenabrowser.net/datapages/). Transcriptome data of 566 colorectal cancer (CRC) samples with clinical information from GSE39582 data set were downloaded from Gene Expression Omnibus (GEO).^11^ RNA sequencing data of 17 382 samples of 30 different types of tissue from 6426 donators were obtained from Genotype‐Tissue Expression (GTEx) project,^12^ and transcriptome of 917 cancer cell line derived from tumour in 24 different sites from Cancer Cell Line Encyclopedia (CCLE)^13^ was obtained through GSE36133.

Tumor Immune Estimation Resource (TIMER) was used for validation of our testing results.^6^, ^14^, ^15^ Additionally, formalin‐fixed, paraffin‐embedded specimen arrays of 79 paired CRC tumour and normal tissues from our centre were used in this study (RJ cohort). Written informed consent was obtained from every enrolled patient. The study protocol was approved by the Ethical Committee of Ruijin Hospital, Shanghai Jiao Tong University Medical School.

### 2.2 Evaluation of neutrophil infiltration

In this study, we use CIBERSORTx^16^ to evaluate the amount of infiltrated neutrophils in tumour tissue. CIBERSORTx is a digital cytometry tool. The algorithm enables researchers to estimate the faction of many kinds of immune cells inside tumour tissue, just by utilizing the gene expression data from bulk tissue, without running an actual flow cytometry. In this research, we evaluated the neutrophils infiltration in CRC tissue using data from TCGA and GSE39582. The calculation results come with a p‐value for each sample. Samples with p‐values < 0.01 were selected for further analysis.

### 2.3 Tumour purity estimation

Tumour purity was evaluated using R package Estimate.^17^ ESTIMATE is a R software for evaluation of tumour purity, stromal cells proportion and the infiltration level of immune cells in tumour tissues based on expression data. In this research, we use ESTIMATE to evaluate the tumour purity of samples in GSE39582 data set.

### 2.4 Co‐expression network analysis and visualization

The WGCNA R software package was used to construct the co‐expression network of the genes in the GSE39582 data set. We selected the top 6000 genes according to the rank of variance (from large to small). A total of 459 tumour samples in the GSE39582 data set were included in the following analysis. After proper samples and genes were chosen, adjacency matrix was constructed by calculating the Pearson correlation between all pairs of genes in all selected samples using the WGCNA R package. Then, the power of power of β = 5 (scale‐free R2 = 0.8970) was used as a soft threshold to ensure a scale‐free network. To further identify functional modules in the co‐expression network with these 6000 genes, the adjacency matrix was used to calculate the topological overlap measurement (TOM) representing the overlap in the shared neighbours.

The module eigengenes (MEs) were considered to be a representation of the gene expression profile in the module. Correlation and P‐value between the module and neutrophils scores were calculated using MEs and neutrophils score.^18^, ^19^ The gene module highly correlated with the neutrophils was selected for visualization using Cytoscape.^20^

### 2.5 Differentially expressed genes (DEGs) and Gene Set Enrichment Analysis (GSEA)

The expression data of TCGA colorectal cancer patient were downloaded and processed using R package DESeq2^21^ to calculate differentially expressed genes. The genes were then ordered according to log2(Foldchange), and the gene list was subjected to gseGO function from clusterProfiler^22^ package for GSEA analysis.

### 2.6 Immunohistochemical staining and histochemistry scoring

CD66b served as specific biomarker for neutrophils in TME. Tissue Microarray sections were deparaffinized and dehydrated, and then treated with 3% H2O2 at room temperature for 10 min to block endogenous peroxidase activity. Next, the tissue sections were incubated with citrate buffer for the retrieval of the antigen. Then, the tissues were blocked with 3% BSA at room temperature for 30 min, followed by incubating with anti‐CD66b(ab197678, rabbit; 1:200, Abcam, Cambridge, UK), anti‐SOD2 antibody (ab13533, Rabbit, 1:100, Abcam, Cambridge, UK) and anti‐CXCL8 antibody(ab18672, Mouse, Abcam, Cambridge, UK) at room temperature for 50min. Finally, the tissue slides were counterstained with haematoxylin. In validation phase, slides were then scanned (Pannoramic MIDI, 3D HISTECH), and intensity of three proteins was evaluated using Quant centre software. The expression level of each genes was quantified as 1, 2 and 3, representing weak, moderate and strong expression, respectively, and then, the histochemistry scores(H‐Score) of SOD2 and CXCL8 of calculated using the method mentioned previously.^23^ Intratumoral neutrophils are identified as CD66b‐positive cells with segmented nuclear. When calculating the correlation coefficient of neutrophil infiltration and genes, the amounts of infiltrated neutrophils were evaluated by the mean density of strong‐positive pixels of CD66b.

### 2.7 Statistics and data visualization

All statistical analyses were performed with R software (version 3.6.2). Wilcoxon test was used to compare the neutrophil scores/counts in different sample groups. Fisher's exact test was used in the categorical clinical traits comparation between the high or low neutrophil group. The Kaplan‐Meier method and log‐rank were used to present and evaluate the differences of survival between the high or low‐neutrophil groups. The cutpoint to determine TINs high/low groups was calculated using ‘surv_cutpoint’ function from R package, survminer.^24^ Data visualization was performed based on ggplot2^25^ and ggpubr ^26^ packages.

## 3 RESULTS

### 3.1 Study design

**FIGURE 1:**
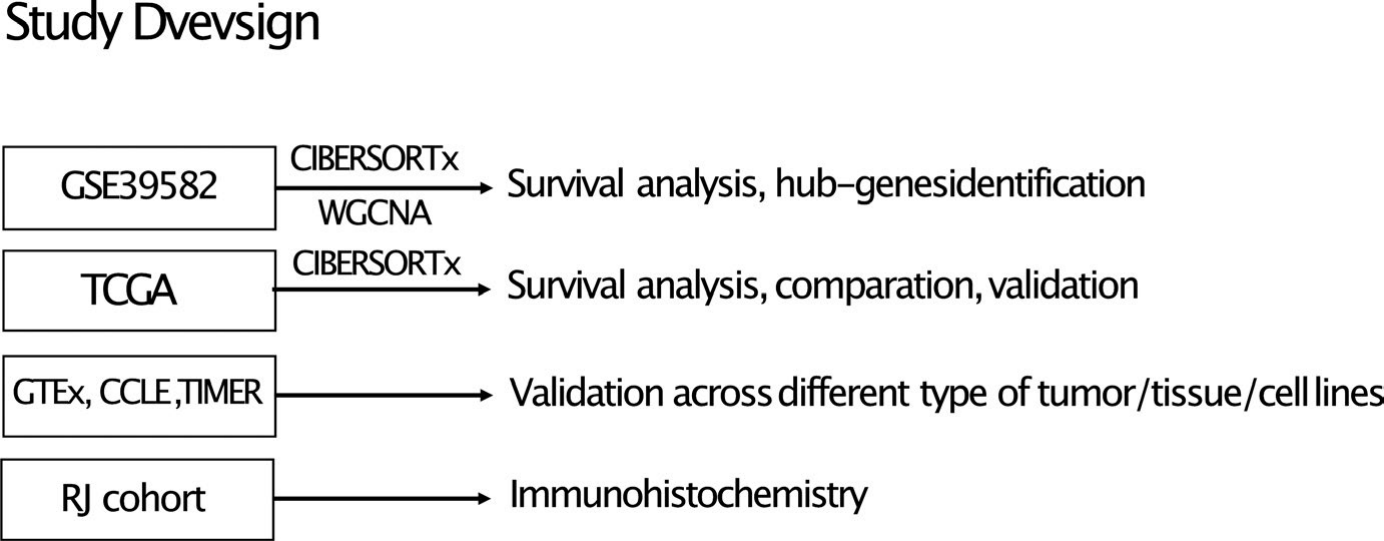
Main design of the study. GSE39682 data set was used to identify hub genes associated with TINs. TCGA COREAD cohort were used for validation, DEGs calculation and GSEA analysis. GTEx, CCLE and TIMER database were used for further validation. RJ cohort were utilized for immunohistochemistry

The design of the study was shown in Figure 1. Gene expression data from GSE39582 (n = 566) and TCGA colorectal patients (n = 603) were subjected to CIBERSORTx to get neutrophils infiltration scores. After identifying hub genes associated with using WGCNA in GSE39582 data set, the correlation between hub genes and neutrophils infiltration was then validated using database including TIMER, GTEx and CCLE. Samples of RJ Cohort from our hospital were used for immunohistochemical validation. In RJ cohort, a total of 79 patients were enrolled, about 59% of the patients were aged over 60 years, 56% of the patients were male (Table S1).

### 3.2 Higher TIN levels indicating poorer prognosis of CRC patients

**FIGURE 2:**
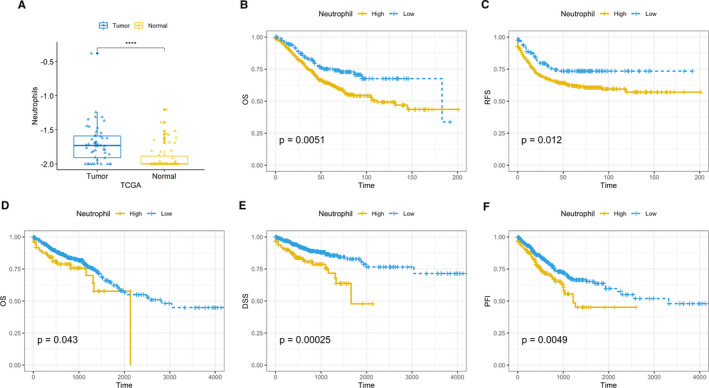
Neutrophils infiltration is higher in tumour tissue and associated with poorer prognosis. A, Neutrophil infiltration score counted by CIBERSORTx in 50 paired tumour‐normal tissue in TCGA COAD and READ cohort. Y‐axis represents log10(neutrophil scores + 0.01). B, Overall survival (OS) in GSE39582 data set. C, Relapse‐free survival (RFS) in GSE39582 data set. D, OS in TCGA COREAD cohort. E, Disease‐specific survival (DSS) in TCGA COREAD cohort. F, Progression‐free interval event (PFI) in TCGA COREAD cohort. ^*^Kaplan‐Meier, log‐rank P‐values were calculated using Wilcoxon tests

As shown, neutrophils score calculated by CIBERSORTx, representing amount of neutrophil infiltration, is significantly higher in tumour tissue compared with normal tissue, indicating an increasing neutrophil recruitment in CRC tumour tissue (Figure 2A). Moreover, higher neutrophil score indicates poorer prognosis in both GSE39582 data set (Figure 2B‐2C) and TCGA data set(Figure 2D‐2F). These findings highlight the clinical relevance of TINs and the necessity to investigate detailed mechanism of neutrophil‐tumour interaction in colorectal cancer.

### 3.3 Neutrophil associated Hub gene identification

Gene expression data from GSE39582 data set were subjected to CIBERSORTx and ESTIMATE algorithm to evaluate neutrophil infiltration and tumour purity. We discovered that tumour purity varies dramatically across samples, arranging from 0.238 to 0.972. As tumour purity might have a great influence on the neutrophil score calculated by CIBERSORTx, the heterogeneity of tumour samples might compromise the robustness of WGCNA. Thus, we filtered out the samples with tumour purity less than 0.5. Also, we discarded samples with p‐values (derived from CIBERSORTx calculation) greater than 0.01. Finally, 459 samples were subjected to WGCNA analysis.

**FIGURE 3:**
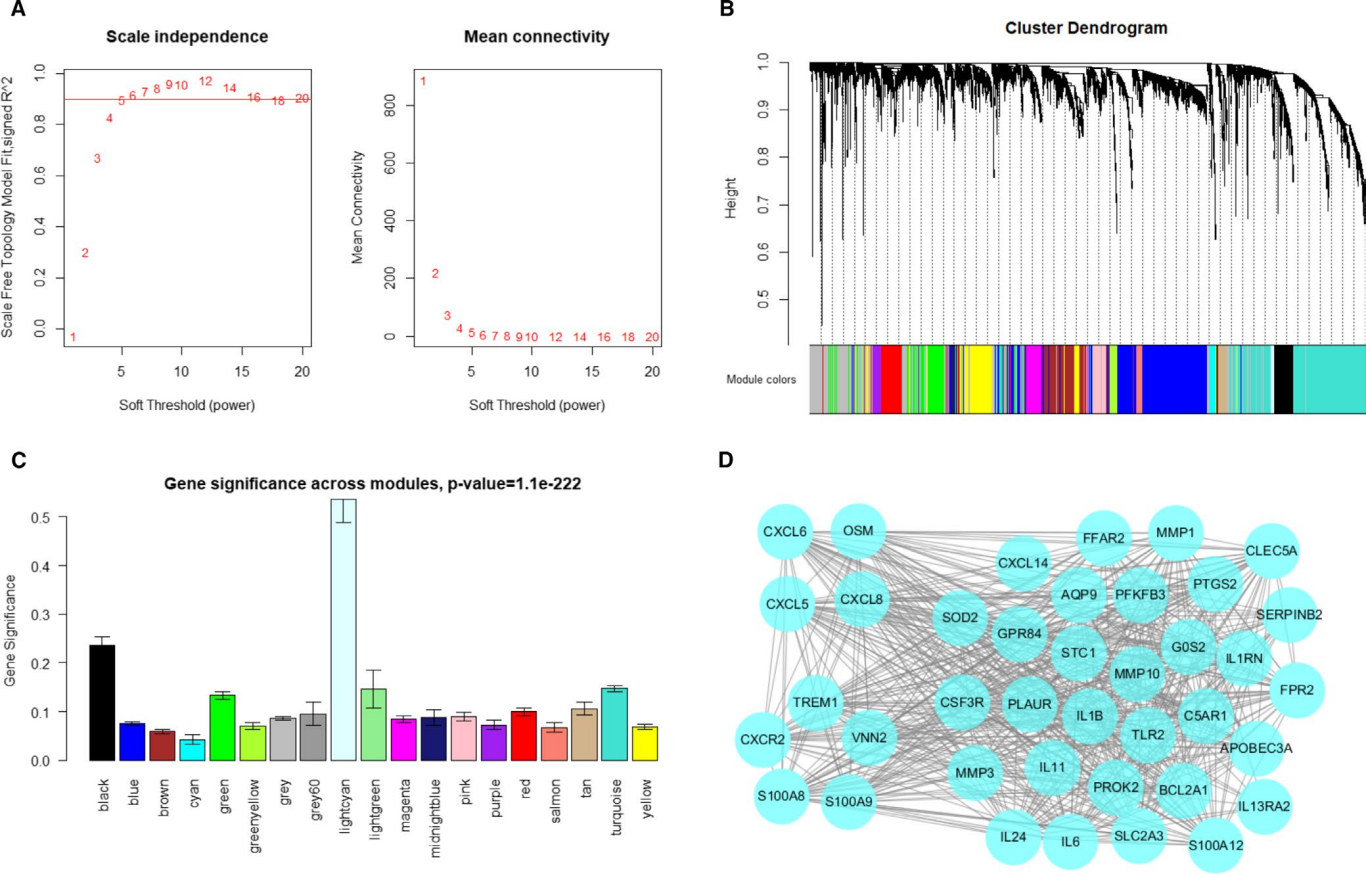
Identifying hub genes associated with neutrophil infiltration in GSE39582 data set. A, Analysis of the scale‐free fit index and the mean connectivity for various soft‐thresholding powers. B, Dendrogram of all differentially expressed genes clustered based on a dissimilarity measure. Dynamic tree cutting was applied to identify modules by dividing the dendrogram at significant branch points. Modules are displayed with different colours in the horizontal bar immediately below the dendrogram. C, Distribution of average gene significance in the modules based on neutrophil score derived from CIBERSORTx. D, Visualization of gene network in lightcyan module

In this study, the power of β = 5 (scale‐free R^2^ = 0.8970) was selected (Figure 3A) to ensure a scale‐free network. A total of 18 gene modules represented by 18 different colours were successfully identified(Figure 3B), in which the lightcyan module was significantly correlated with neutrophil score (Figure 3C). Genes in lightcyan module were exported and visualized in Cytoscape (Figure 3D). As expected, genes highly expressed in neutrophils including S100A8, S100A9, VNN2 and TERM1 are included in the module. Besides, three C‐X‐C Motif Chemokine Ligand members, CXCL5, CXCL6, CXCL8, were also included. These results reflected the robustness of WCGNA.

Theoretically, most of the genes in lightcyan module should be correlated with neutrophil infiltration and neutrophil‐attract chemokines, regardless of the expression level. In order to facilitate the immunohistochemical analysis, we decided to choose a relatively highly expressed gene from this module, SOD2 (Figure S1), for further analysis. Intriguingly, the other members of SOD family, SOD1 and SOD3, are not correlated with neutrophils infiltration. This is an indicator that the correlation between SOD2 and TINs might be irrelevant to oxidative stress induced by neutrophils. Thus, we chose SOD2 for further analysis.

### 3.4 Validation of SOD2 associated TME neutrophil infiltration in TCGA and RJ cohort

**FIGURE 4:**
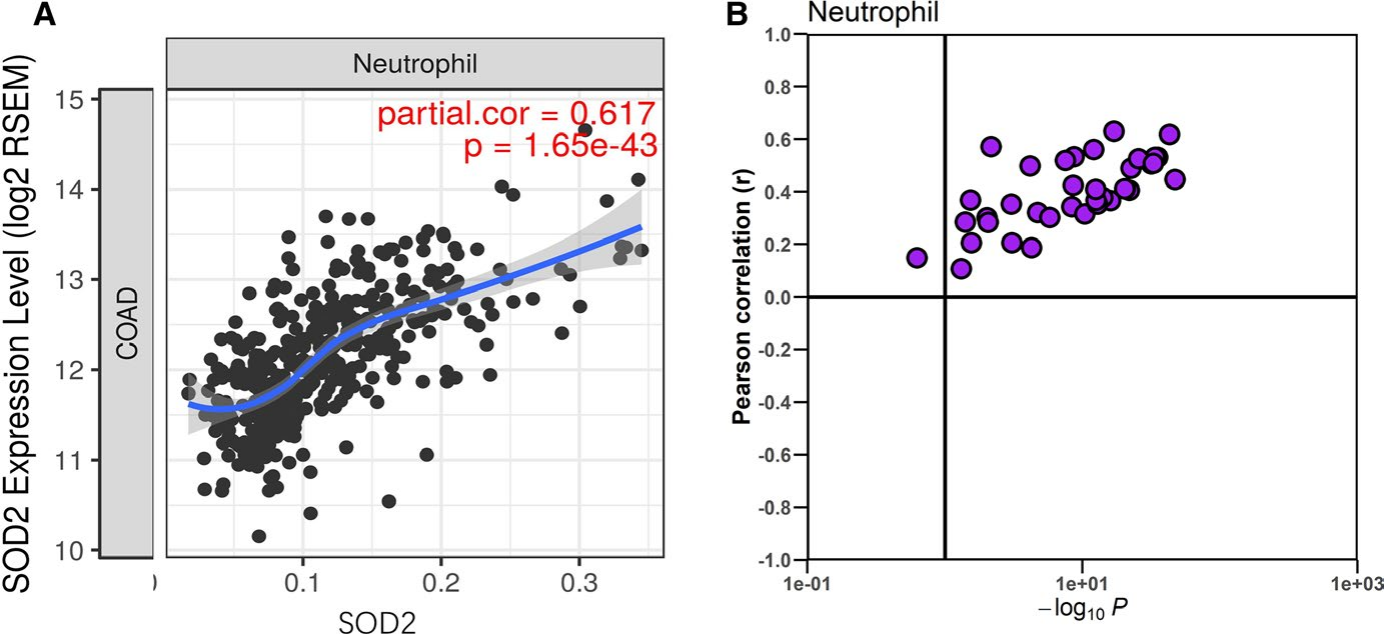
Association of SOD2 and neutrophils in TCGA cohort. A, Correlation of SOD2 and TINs in TCGA COAD cohort. B, Correlation of SOD2 and TINs across 32 different type of cancer. The vertical line corresponds to a p‐value of 0.1

In order to validate the association of SOD2 and neutrophil infiltration, we firstly tested our findings in TCGA cohort using TIMER. As shown, the expression of SOD2 in CRC tumour tissue is significantly correlated with neutrophils infiltration in CRC tissue (Figure 4A). We further explored the association in 32 different kinds of solid tumours. Intriguingly, SOD2 is related to TINs in most of the cancer types(Figure 4B). This finding indicated a universal role SOD2 in neutrophils infiltration in TME. Intriguingly, SOD1 and SOD3 are not substantially correlated with TINs (Figure S2).

**FIGURE 5:**
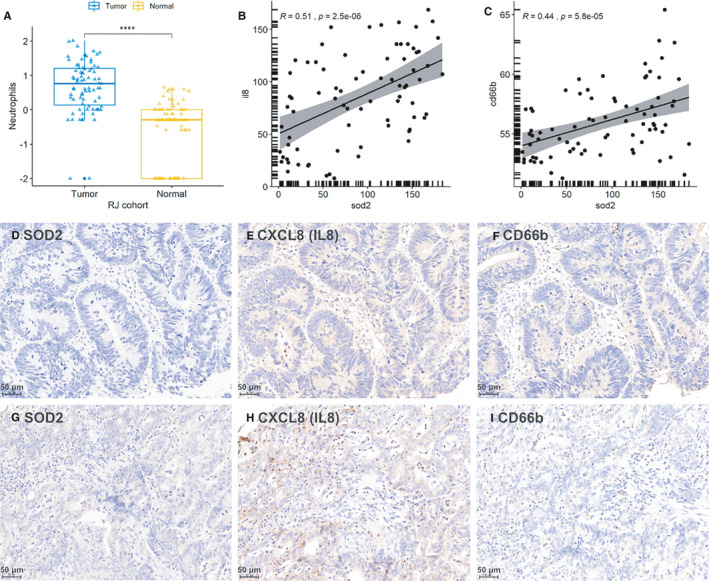
Validation of SOD2‐CXCL8‐Neutrophil infiltration association in RJ cohort. A, Neutrophils counts in tumour tissue and normal tissue in RJ cohort. Y‐axis represents log10(neutrophil count + 0.01). B, Pearson correlation of SOD2‐CXCL8(IL8) association in RJ cohort (expression levels are quantified by H‐score). C, Pearson correlation of SOD2‐CD66b association in RJ cohort (expression levels are quantified by H‐score). D‐F, Representative images of low SOD2 expression, low CXCL8 expression and low neutrophil infiltration in the same tissue region, respectively. G‐I, Representative images of high SOD2 expression, high CXCL8 expression and high neutrophil infiltration in the same tissue region, respectively

For further verifying, we conducted immunohistochemistry analysis in RJ cohort, which contains 79 cases of CRC patients. As shown (Figure S3), cells positive for CD66b antibody and with segmented nuclear are identified as neutrophils. As expected, neutrophil count in tumour tissue are significantly higher than that in normal colon tissue(Figure 5A). Expression levels of SOD2 are positively correlated with CXCL8 (Figure 5B, Pearson correlation, R = 0.51, *P* <.001) and neutrophil infiltration (Figure 5C, Pearson correlation, R = 0.44, *P* <.001), as shown in representative images (Figure 5D‐5I).

### 3.5 Functional investigation of downstream signalling associated with SOD2 expression

**FIGURE 6:**
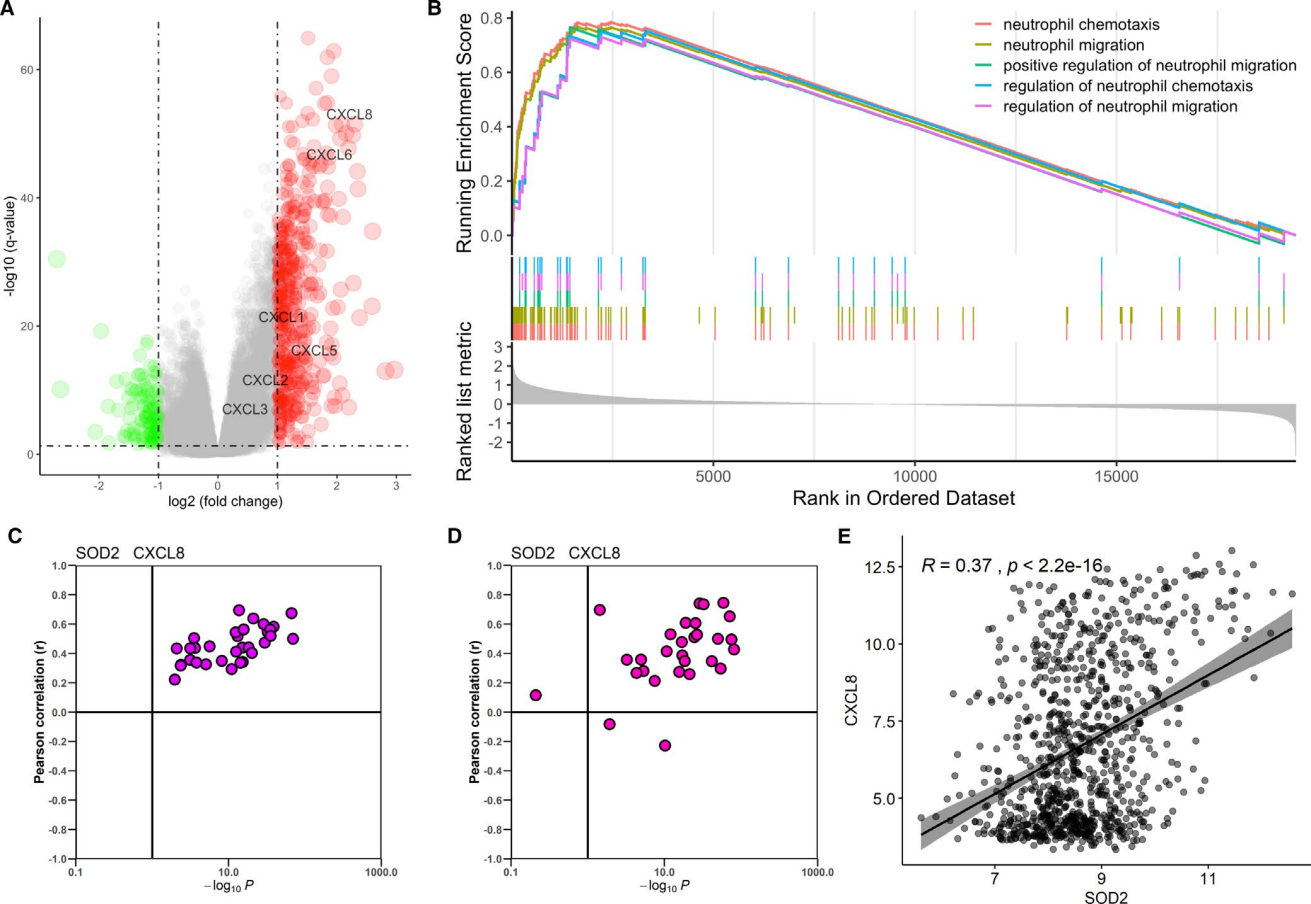
Functional exploration of SOD2. A, Volcano plot of Differentially expressed genes (DEGs) of high/low SOD2 group in TCGA COARD cohort. B,GSEA analysis, genes are ordered by logFC value derived from DEGs calculation. C, Correlation of SOD2 and CXCL8 in 32 types of cancer from TCGA data. Vertical line corresponding with a p‐value of 0.1. D, Correlation of SOD2 and CXCL8 in 30 types of tissue from GTEx data. Vertical line corresponding with a p‐value of 0.1. E, Correlation of SOD2 and CXCL8 in 917 cancer cell lines from CCLE

TCGA colorectal cohort was used to further investigate the SOD2 related biological activity. Based on the normalized expression level of SOD2, we divided the samples into 2 groups, with 312 and 313 samples in SOD2 high and low groups, respectively. Differentially expressed genes were calculated using DESeq2 package. As shown in the volcano plot (Figure 6A), we found that CXCL6 and CXCL8 expression levels are significantly higher in SOD2 high group comparing to low group. Gene Set Enrichment Analysis (GSEA) of GO biological process indicates that immunological differences concerning neutrophils recruitment are significantly higher in SOD2 high group (Figure 6B). These findings indicate that a SOD2 involved neutrophil recruitment regulating axis might exist.

As CXCL1, CXCL2, CXCL5, and CXCL8 are frequently reported chemokines to recruit neutrophils, we set our course to explores the expressional correlation between SOD2 and these chemokines. The correlation of SOD2 and the four chemokines were evaluated in CCLE, GTEx and TCGA gene expression data. As shown, the SOD2 are significantly correlated with these chemokines, especially CXCL8, in most of the cancer types and normal tissues (Figure 6C‐6E, Figure S4), indicating a universal association between SOD2 and CXCL8.

These results showed that a ‘SOD2‐CXCL8‐Neutrophil infiltration’ axis might exists in CRC. However, detailed mechanisms by which SOD2 orchestrating neutrophils infiltration needs further investigation.

## 4 DISCUSSION

Due to the success of immune checkpoint inhibitors (ICIs) therapy in clinical trial, CD8 + T cells are put under spotlight in recent years.^3^ Although emerging cases showing the efficacy of immunotherapy, a large proportion of patients were not responsive to ICIs. Thus, studies aiming at exploiting alternative therapeutic targets are necessary. Neutrophils, as the first responders of infection and inflammation, have been neglected by tumour biologists for a long time. Recent years, researchers started to pay attention to the role of neutrophils played in cancer progression.^27^

Neutrophils are usually considered as first‐line responder against extracellular pathogens. Just as tumour‐associated macrophages (TAMs) being divided into ‘M1’ anti‐tumour phenotype and ‘M2’ pro‐tumour phenotype, emerging evidence exhibit that TINs can polarize into ‘N1’ anti‐tumour phenotype or ‘N2’ pro‐tumour phenotype. In fact, transforming growth factor beta (TGF‐β) promote neutrophil differentiation into N2 type, whereas interferon type I (IFN‐I) induces N1 type. In a word, the signalling context in the tumour microenvironment can affect the phenotype of neutrophils profoundly.

The increase of neutrophil count in peripheral circulation are found to related to poorer prognosis in CRC patients.^28^, ^29^ However, the effect of TINs in CRC patients has been controversial. Rao et al reported that increase in TINs is related to are predictor of poor prognosis in CRC patients,^30^ whereas Berry at al. showed that high levels of TINs were associated with improved overall survival.^31^

Several experiments demonstrated that CRC cells recruit neutrophils by secreting chemokines including CCL‐9, CCL15, CXCL8.^32^, ^33^ Neutrophils also promote the hepatic metastasis of CRC, whereas depletion of neutrophils in murine decreased the vascular density and branching in metastatic site and inhibited the tumour growth.^34^ Intriguingly, in CRC patients receiving anti‐VEGF (bevacizumab) therapy, the present of CD177 + neutrophils in tumour is related to reduced survival, and depletion of neutrophils restores the sensitivity of anti‐VEGF treatment.^35^

Tohme, S et al reported that neutrophil extracellular traps (NETs), complexes of chromosomal DNA, histones and other proteins released by neutrophils, can release High Mobility Box (HMGB)‐1 protein and activate of TLR9‐dependent pathways in CRC cells to promote their adhesion, proliferation, migration and invasion, thus facilitating liver metastasis.^36^

T cell infiltration in CRC tissue is associated with favourable outcome. As neutrophils frequently colocalize with CD8 + Tcells,^37^ the crosstalk between TINs and T cells intrigued researchers in recent years. Due to the plasticity of TINs, the result of neutrophil‐T cell interaction in CRC can be controversial. Governa V et al reported that CD8 + T cell activation is enhanced when cocultured with neutrophils in vitro, and combined infiltration of CD66b + neutrophils and CD8 + T lymphocytes in CRC is associated with significantly better survival comparing to CD8 + T cell infiltration alone.^37^ However, Neutrophil‐derived MMP9 activates TGF‐β and consequently induced T cell suppression in CRC.^38^ Zhang Y demonstrated that neutrophils selectively deprived of IRAK‐M or Tollip significantly activate T cells by enhanced expression of the costimulatory molecule CD80, and reduced expression of the inhibitory molecule PD‐L1^39^, ^40^ indicating modified neutrophils can be a promising therapeutic strategy in CRC treatment.

Despite the difficulties in TINs investigation, the accumulating evidence is showing the substantial role of neutrophils in tumour initiation and metastasis.^41^ This finding raised the hope of inhibiting cancer development by targeting TINs. Thus, it is imperative to elucidate the molecular mechanism regulating the neutrophils and cancer cells interaction. In this study, we found that low neutrophils infiltration in CRC were associated with the better prognosis. With the help of bioinformatic analytical tools, a list of genes highly associated with TINs in colorectal cancer were identified. We then selected SOD2 among all of the genes to conduct a further analysis. The significant correlation of SOD2, CXCL8 and neutrophils infiltration are validated immunohistochemically in both TCGA cohort and RJ cohort.

SOD2 is a member of superoxide dismutase family, located in mitochondria. This gene is known as a scavenger of mitochondrial derived ROS. As ROS are considered to be a crucial regulator of cell signalling network that drive proliferation and malignant progression, the superoxide dismutase plays a putatively crucial role in tumorigenesis. Interestingly, we found that SOD2 is positively correlated with TINs infiltration in most of the cancer type, indicating a universal association between SOD2 and neutrophil in TME. Correspondingly, we found that SOD2 is associated with several neutrophil‐attract chemokines. As neutrophils carries cytotoxic materials including H_2_O_2_, whether the high expression of SOD2 in colorectal cancer is induced by ROS stress derived from neutrophils still needs to be elucidated. Of note, SOD1 and SOD3 have no such association with neither neutrophils nor related chemokines, indicating additional functions of SOD2 in tumour progression except for ROS scavenging.

Altogether, we propose that a ‘SOD2‐CXCL8‐neutrophil recruitment’ axis might exist in CRC cells. Our analysis provided insights into the molecular mechanism of crosstalk between neutrophils and tumour, which may facilitate further investigation of TINs in the near future.

## 5 CONCLUSION

In summary, based on the comprehensive analysis of transcriptome data using Cibersort and WGCNA algorism, we discovered a list of genes highly associated with TINs. Among which, SOD2 significantly correlated with neutrophil infiltration and neutrophil‐related chemokines in CRC, indicating a possible existence of ‘SOD2‐CXCL8‐neutrophil recruitment’ in CRC. The findings of our study may facilitate the further research in crosstalk between neutrophil and colorectal cancer cells.

## CONFLICT OF INTEREST

The authors declare that there is no conflict of interest.

## AUTHOR CONTRIBUTION

**Hao Su:** Conceptualization (equal); Investigation (equal); Methodology (equal); Software (equal); Writing‐original draft (equal). **Tianyi Cai:** Validation (equal); Visualization (equal); Writing‐original draft (equal). **Sen Zhang:** Writing‐review & editing (equal). **Xialin Yan:** Software (equal). **Leqi Zhou:** Methodology (equal). **Zirui He:** Conceptualization (equal). **Pei Xue:** Resources (equal). **Jianwen Li:** Resources (equal). **MinHua Zheng:** Supervision (equal). **Xiao Yang:** Project administration (equal); Supervision (equal); Visualization (equal). **Bo Feng:** Funding acquisition (equal); Methodology (equal); Project administration (equal); Resources (equal); Supervision (equal); Writing‐review & editing (equal).

## ETHICAL STATEMENT

Written consent was obtained from all enrolled patients. The study was conducted in accordance with the Declaration of Helsinki, and the Ethic Committee of RuiJin Hospital, Shanghai Jiao Tong University approved the protocol of the study (GCQN‐2019‐A07).

## Supporting information

Figure S1

Figure S2

Figure S3

Figure S4

Table S1

## Data Availability

The RNA sequencing data of CRC patients were downloaded from Xena (http://xena.ucsc.edu/). GSE39582 and GSE36133 were obtained from GEO database (https://www.ncbi.nlm.nih.gov/gds/). Other data analysed during this study are included in this published article and the supplementary material.
