# Supplementary figures and images for: Identification of hub genes associated with neutrophils infiltration in colorectal cancer
Source: J Cell Mol Med. 2021 Mar 5;25(7):3371–80. doi: 10.1111/jcmm.16414 (PMC8034475; doi:10.1111/jcmm.16414)

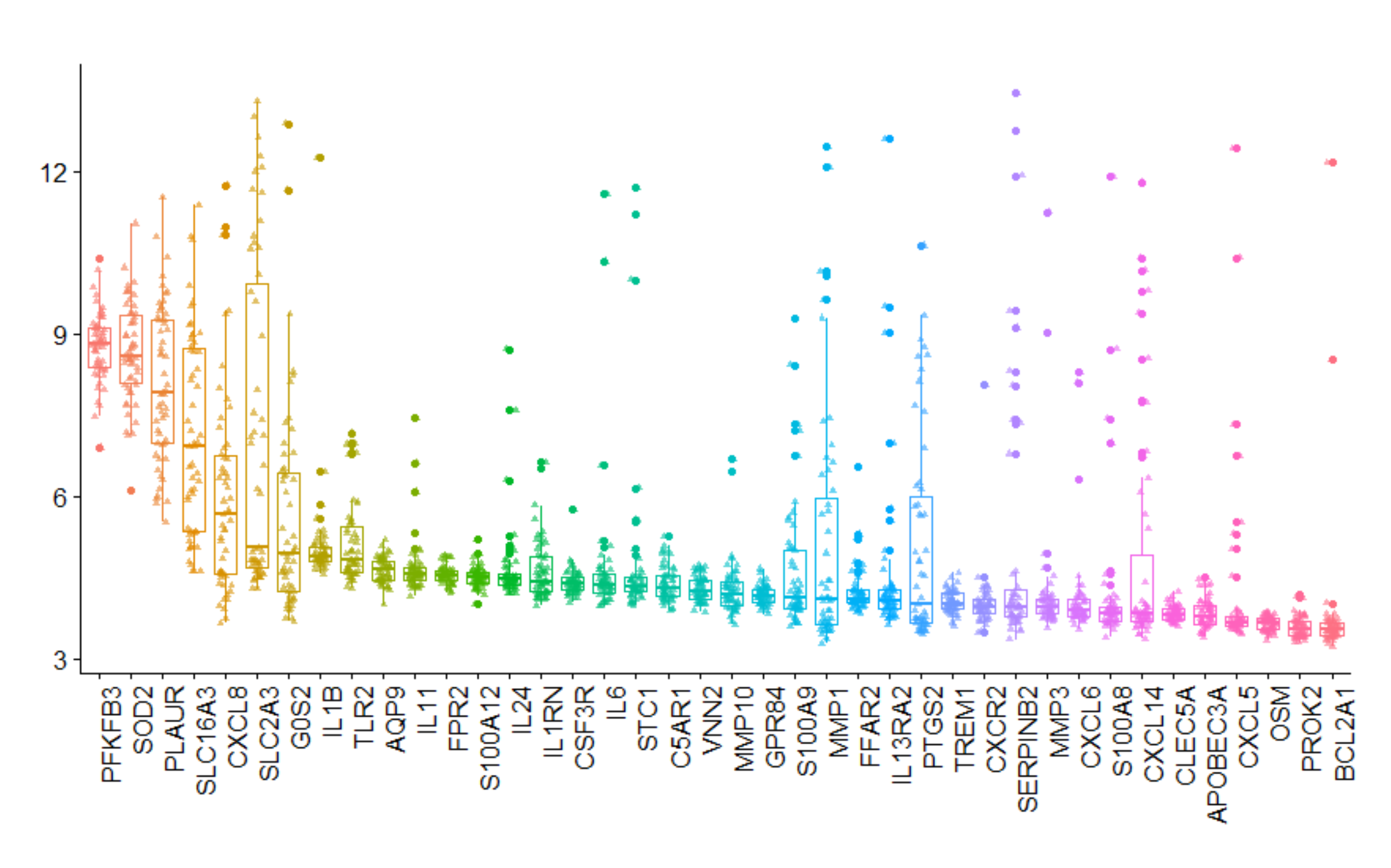

Supplement: Supplementary file 1 — Figure S1 [file JCMM-25-3371-s001.tiff]

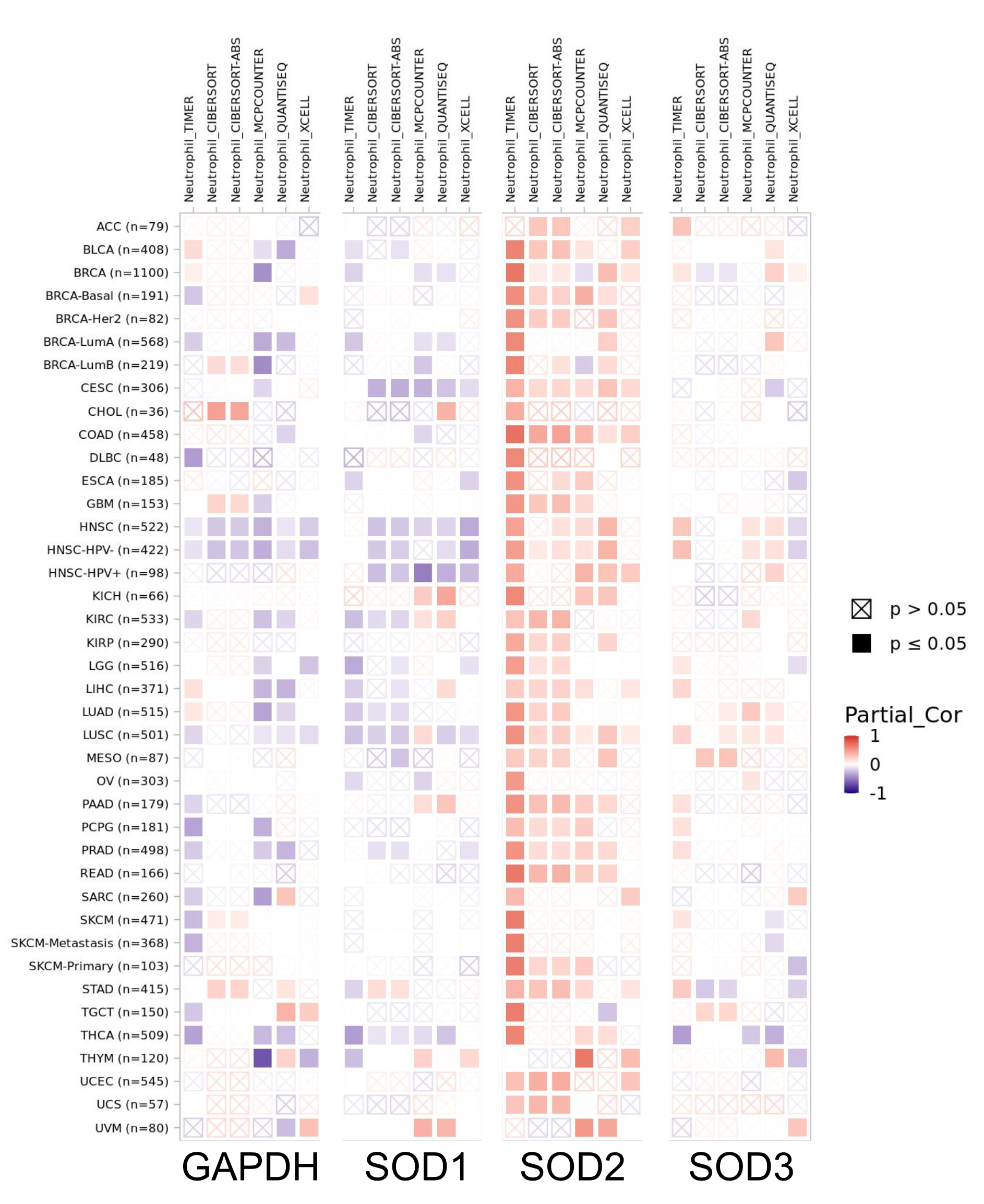

Supplement: Supplementary file 2 — Figure S2 [file JCMM-25-3371-s002.tiff]

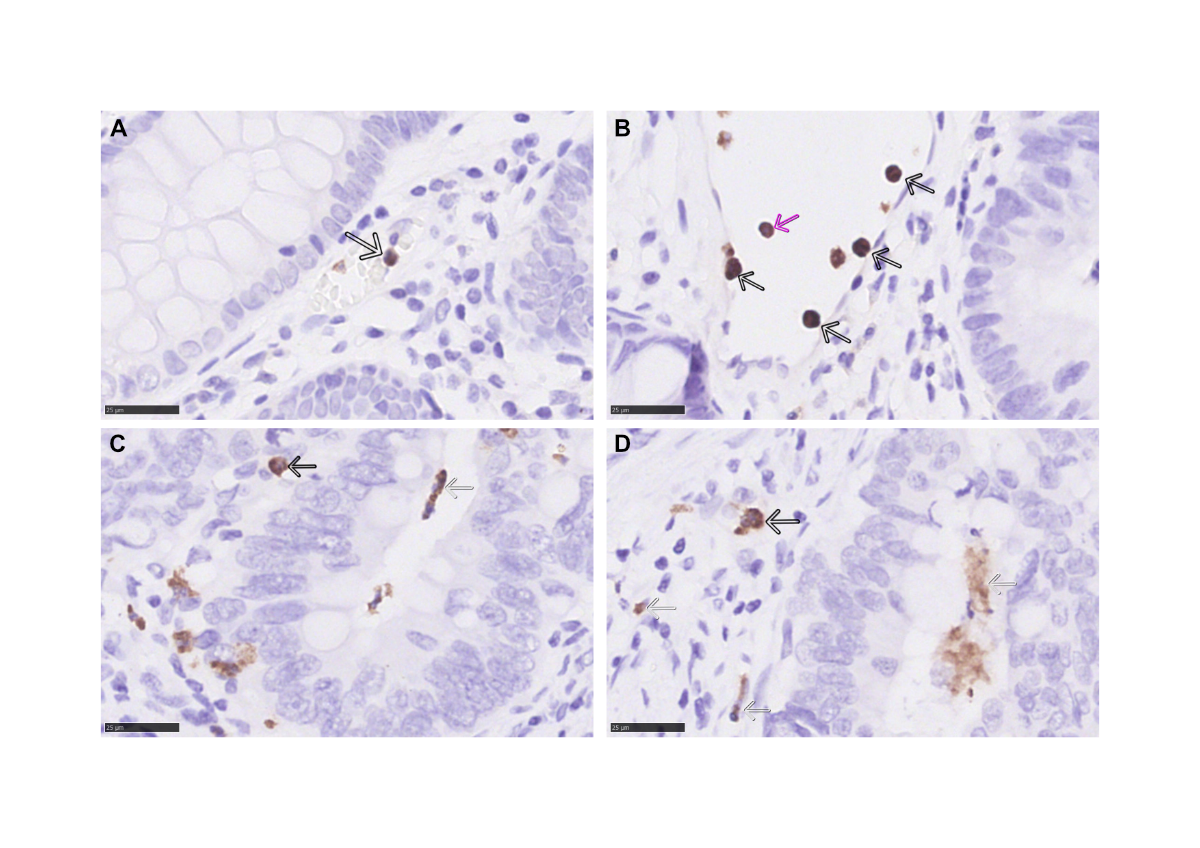

Supplement: Supplementary file 3 — Figure S3 [file JCMM-25-3371-s003.tiff]

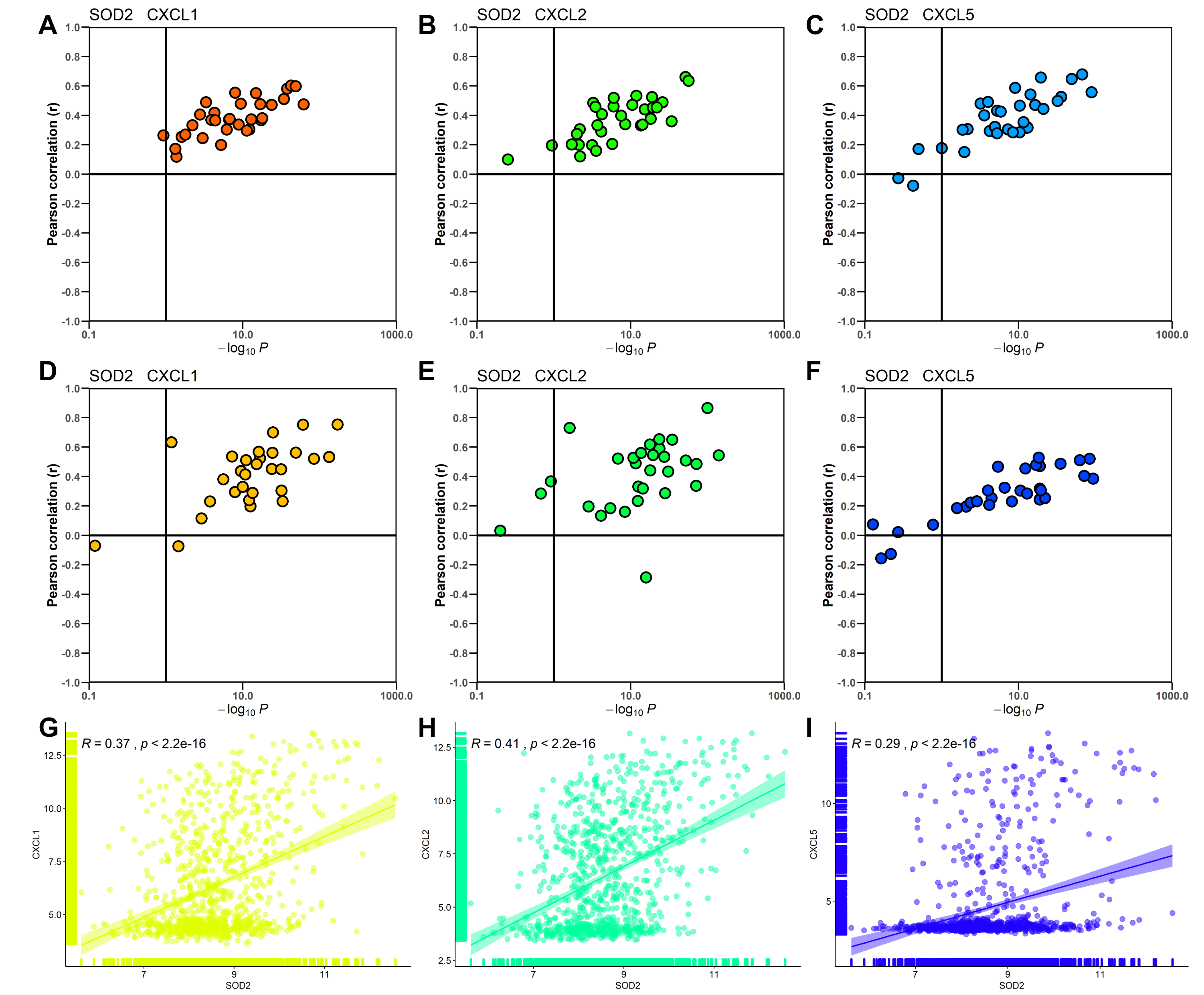

Supplement: Supplementary file 4 — Figure S4 [file JCMM-25-3371-s005.tiff]
