# Supplementary material for: Identification of hub genes associated with neutrophils infiltration in colorectal cancer
Source: J Cell Mol Med. 2021 Mar 5;25(7):3371–80. doi: 10.1111/jcmm.16414 (PMC8034475; doi:10.1111/jcmm.16414)
Supplement: Supplementary file 5 — Table S1 [file JCMM-25-3371-s004.doc]

Table.S1 Clinical characteristics of CRC patients from Ruijin Cohort (n=79).

| variate | group | | p |
| --- | --- | --- | --- |
|  | High (n = 57) | Low (n = 22) |  |
| Gender (%) |  |  | 1 |
| 0 | 24 (42.9) | 10 (45.5) |  |
| 1 | 32 (57.1) | 12 (54.5) |  |
| Age (%) |  |  | 0.156 |
| <60 | 19 (33.9) | 12 (54.5) |  |
| ≥60 | 37 (66.1) | 10 (45.5) |  |
| Tumor.location (%) |  |  | 1 |
| distal | 39 (69.6) | 16 (72.7) |  |
| proximal | 17 (30.4) | 6 (27.3) |  |
| T (%) |  |  | 0.564 |
| T1+T2 | 15 (26.3) | 4 (18.2) |  |
| T3+T4 | 42 (73.7) | 18 (81.8) |  |
| N (%) |  |  | 0.805 |
| N0 | 33 (57.9) | 12 (54.5) |  |
| N1+N2 | 24 (42.1) | 10 (45.5) |  |
| M (%) |  |  | 1 |
| 0 | 54 (94.7) | 21 (95.5) |  |
| 1 | 3 (5.3) | 1 (4.5) |  |
|  |  |  | 1 |
| dMMR | 6 (10.5) | 2 (9.5) |  |
| pMMR | 51 (89.5) | 19 (90.5) |  |
| KI67 (%) |  |  | 0.381 |
| <0.6 | 5 (8.8) | 4 (19.0) |  |
| >0.9 | 10 (17.5) | 2 (9.5) |  |
| 0.6~0.9 | 42 (73.7) | 15 (71.4) |  |
| Grade (%) |  |  | 0.586 |
| 1-2 | 40 (70.2) | 13 (61.9) |  |
| 3 | 17 (29.8) | 8 (38.1) |  |

*Fisher exact test*
